# Single-Cell Analysis of Bone Marrow CD8^+^ T Cells in Myeloid Neoplasms Reveals Pathways Associated with Disease Progression and Response to Treatment with Azacitidine

**DOI:** 10.1158/2767-9764.CRC-24-0310

**Published:** 2024-12-04

**Authors:** Athanasios Tasis, Nikos E. Papaioannou, Maria Grigoriou, Nikolaos Paschalidis, Catherine Loukogiannaki, Anastasia Filia, Kyriaki Katsiki, Eleftheria Lamprianidou, Vasileios Papadopoulos, Christina Maria Rimpa, Antonios Chatzigeorgiou, Ioannis Kourtzelis, Petroula Gerasimou, Ioannis Kyprianou, Paul Costeas, Panagiotis Liakopoulos, Konstantinos Liapis, Petros Kolovos, Triantafyllos Chavakis, Themis Alissafi, Ioannis Kotsianidis, Ioannis Mitroulis

**Affiliations:** 1 Translational Research and Laboratory Medicine Unit, First Department of Internal Medicine, University Hospital of Alexandroupolis, Democritus University of Thrace, Alexandroupolis, Greece.; 2 Department of Hematology, University Hospital of Alexandroupolis, Democritus University of Thrace, Alexandroupolis, Greece.; 3 Laboratory of Immune Regulation, Center of Basic Sciences, Biomedical Research Foundation Academy of Athens, Athens, Greece.; 4 Laboratory of Autoimmunity and Inflammation, Center of Clinical, Experimental Surgery and Translational Research, Biomedical Research Foundation Academy of Athens, Athens, Greece.; 5 Department of Physiology, Medical School, National and Kapodistrian University of Athens, Athens, Greece.; 6 Hull York Medical School, York Biomedical Research Institute, University of York, York, United Kingdom.; 7 Molecular Hematology-Oncology, Karaiskakio Foundation, Nicosia, Cyprus.; 8 Department of Molecular Biology and Genetics, Democritus University of Thrace, Alexandroupolis, Greece.; 9 Institute for Clinical Chemistry and Laboratory Medicine, University Hospital and Faculty of Medicine Carl Gustav Carus of TU Dresden, Dresden, Germany.; 10 National Center for Tumor Diseases, Partner Site Dresden, Dresden, Germany.; 11 Laboratory of Biology, School of Medicine, Athens, Greece.

## Abstract

CD8^+^ T cells are crucial for antitumor immunity. However, their functionality is often altered in higher-risk myelodysplastic neoplasms (MDS) and acute myeloid leukemia (AML). To understand their role in disease progression, we conducted a comprehensive immunophenotypic analysis of 104 pretreatment bone marrow (BM) samples using mass and flow cytometry. Our findings revealed an increased frequency of CD57^+^CXCR3^+^ subset of CD8^+^ T cells in patients who did not respond to azacitidine (AZA) therapy. Furthermore, an increased baseline frequency (>29%) of the CD57^+^CXCR3^+^CD8^+^ T-cell subset was correlated with poor overall survival. We performed single-cell RNA sequencing to assess the transcriptional profile of BM CD8^+^ T cells from treatment-naïve patients. The response to AZA was linked to an enrichment of IFN-mediated pathways, whereas nonresponders exhibited a heightened TGF-β signaling signature. These findings suggest that combining AZA with TGF-β signaling inhibitors targeting CD8^+^ T cells could be a promising therapeutic strategy for patients with higher-risk MDS and AML.

**Significance:**

Immunophenotypic analysis identified a BM CD57^+^CXCR3^+^ subset of CD8^+^ T cells associated with response to AZA in patients with MDS and AML. Single-cell RNA sequencing analysis revealed that IFN signaling is linked to the response to treatment, whereas TGF-β signaling is associated with treatment failure, providing insights into new therapeutic approaches.

## Introduction

Myelodysplastic neoplasms (MDS), chronic myelomonocytic leukemia (CMML), and acute myeloid leukemia (AML) are clonal disorders that share common pathobiology features (1). Although genetic alterations and epigenetic modifications are central to the pathobiology of myeloid neoplasms, the interaction between clonal and immune cells is also crucial (2–4). Compelling evidence indicates that the interplay between clonal cells and the bone marrow (BM) microenvironment plays a significant role in the development and progression of MDS and CMML (5, 6). CD8^+^ T cells play a crucial role in the regulation of the tumor microenvironment (7). The aberrant functionality of CD8^+^ T cells has been observed in patients with myeloid neoplasms compared with healthy individuals (4, 8, 9), rendering this cell population a major target for immunotherapeutic interventions (10, 11).

The standard of care for patients with higher-risk MDS (HR-MDS) and non-proliferating CMML, as well as for patients with secondary AML who are not eligible for intensive chemotherapy, is treatment with hypomethylating agents (HMA) such as azacitidine (AZA; refs. 12–14). Additionally, AZA along with venetoclax is currently used in patients with previously untreated AML (15). However, not all patients exhibit a favorable response, and there is a significant risk of relapse (16). Additionally, molecular predictors of the response to HMA and the precise mechanism of action of this drug are not well defined. The exact genetic and cellular processes through which HMA exerts its effects are still being studied (13). However, no therapeutic approach can overcome resistance to treatment (17). Several lines of evidence suggest that AZA promotes cellular and cytokine-mediated effector T-cell tumor lysis (18, 19). Nevertheless, the exact role of CD8^+^ cells in disease progression and the response to HMA in myeloid neoplasms remains unclear.

This study aimed to comprehensively investigate the immune cell compartment in the BM of patients with myeloid neoplasms at various disease stages to identify immune cell populations or molecular pathways that could predict treatment response to HMAs or serve as targets for immunotherapy. We initially conducted a systematic analysis of the BM immune landscape using mass cytometry [cytometry by time of flight; (CyTOF)] to immunophenotype diverse immune cell populations. Building on these findings, we then focused on BM CD8^+^ T cells and performed single-cell RNA sequencing (scRNA-seq) to identify molecular signatures in CD8^+^ T-cell subsets that correlate with response to AZA therapy.

## Materials and Methods

### Study design

The primary aim of our study was to investigate the BM immune landscape in patients with myeloid neoplasms, with the goal of identifying specific immunophenotypic differences and molecular signatures associated with disease progression and patient response to AZA treatment. To achieve this, we first used mass cytometry (CyTOF) to analyze immune cell populations in BM samples from patients with lower-risk MDS (LR-MDS), higher-risk MDS (HR-MDS), CMML, and AML before treatment. We then used flow cytometry in an independent cohort to validate the CyTOF results. Building on our immunophenotypic findings, we focused on BM CD8^+^ T cells and performed scRNA-seq to determine the molecular signatures of CD8^+^ T-cell subsets.

### Study patients

BM samples were collected from treatment-naïve patients with MDS, AML, and CMML-2. Patient diagnosis was based on the 2022 fifth World Health Organization classification, (20) and patients with MDS were categorized based on the Revised International Prognostic Scoring System (IPSS-R score) into LR-MDS (IPSS-R score ≤3.5) and HR-MDS (IPSS-R score >3.5; ref. 21). Except for patients with LR-MDS, the patients were treated with AZA at a subcutaneous dose of 75 mg/m^2^ for 7 consecutive days within a 28-day cycle. To manage myelotoxicity or complications associated with myelosuppression, potential measures such as reducing the dose by up to 50% or delaying treatment were considered. Response assessment was performed using the International Working Group Response Criteria for MDS (22) and the recently revised European LeukemiaNet criteria for AML (23). The baseline characteristics of the patients included in this study are provided in Supplementary Table S1. The study was approved by the local Ethics Committee, under the reference number (877). All patients provided informed written consent in accordance with the principles outlined in the Declaration of Helsinki.

### Collection and handling of samples

Density gradient centrifugation, using Ficoll-Histopaque 1077 (Sigma-Aldrich), was employed to isolate bone marrow mononuclear cells (BMMCs). Immediately after isolation, BMMCs were cryopreserved in a freezing medium consisting of 90% FBS and 10% DMSO.

### Mass cytometry and data analysis

High-dimensional immunophenotyping of BMMCs was performed using mass cytometry using established and validated workflows from previous studies (24, 25). We employed the Maxpar Direct Immune Profiling Assay, which contains 30 preconjugated antibodies (Supplementary Table S2) with metal probes in lyophilized form (Standard BioTools; ref. 26). Before staining, BMMCs were thawed in prewarmed RPMI medium supplemented with 10% FBS. After two washes, the cells were resuspended in fresh medium. BMMCs were subjected to a blocking step using Human TruStain FcX (BioLegend; RRID: AB_2818986). Subsequently, cells were stained for surface markers following the Maxpar Direct Immune Profiling Assay manufacturer’s instructions. Two additional washes with Cell Staining Buffer were performed, and fixation was carried out using a 1.6% filtered formaldehyde solution (Sigma) for 20 minutes at room temperature. Finally, cells were stained in a DNA intercalator solution (1:1,000 dilution of 125 μmol/L Cell-ID Intercalator-Ir) in Maxpar Fix and Perm buffer (Standard BioTools). The next day, cells were washed with Cell Staining Buffer and Cell Acquisition Solution and then resuspended with EQ Passport beads (Standard BioTools, 1:10 dilution) immediately before acquisition. Acquisition was performed using a Helios system. To ensure data quality during acquisition, the flow rate in the Helios system (RRID: SCR_019916) did not exceed 350 events per second. Data were subsequently normalized using Passport beads with CyTOF software (version 10.7.1014). Before analysis, we performed data cleanup using bivariate dot plots in FlowJo (v10.8 Software, BD Biosciences; RRID: SCR_008520) to refine Gaussian parameters, and live, singlet cell events were selected for downstream analysis. Data analysis was performed on CD45^+^ cells to exclude blasts from the analysis. FlowSOM (RRID: SCR_016899) clustering analysis (version 2.11.2), dimensionality reduction via t-distributed stochastic neighbor embedding (t-SNE; Rtsne, version 0.17; RRID: SCR_024305), and uniform manifold approximation and projection (UMAP; uwot, version 0.2.2; RRID: SCR_018217), which were implemented within the CATALYST (version 1.26.1; RRID: SCR_017127) package, were performed out in the R programming environment (version 4.1.0), following established open-source workflows previously described (25).

For targeted analysis of T-cell populations, data were imported into Cytobank (accessible at https://premium.cytobank.org; RRID: SCR_014043) for further assessment. All related statistical tests and illustrations were generated using Cytobank. The FlowSOM algorithm (version 2.11.2) was used to hierarchically cluster gated CD4^+^ and CD8^+^ T-cell populations into distinct metaclusters based on their surface marker expression profiles. Proportional sampling was used to maximize the inclusion of all events in the analysis. The default/automatic settings were used for the clustering method, iterations, seed, and number of clusters, whereas the number of metaclusters was set to 10 or 6 based on the specific analysis requirements. Metaclusters, were also projected onto representative t-SNE maps generated using the dimensionality reduction algorithm t-SNE-CUDA using the default parameters provided by Cytobank.

### Flow cytometry

Sample preparation and flow cytometry was performed as previously described (18). Briefly, cryopreserved BMMCs were thawed in a water bath (37°C) and washed with PBS. Following a centrifugation at 300× *g* for 10 minutes, the supernatant was carefully discarded, and the cell pellet was resuspended in PBS. The cells were then treated with DNAse I 1 mg/mL (Sigma-Aldrich) for 10 minutes at room temperature. Next, the BMMCs were stained with a custom antibody panel for 20 minutes, on ice. Details about the antibodies used are provided in Supplementary Table S3. The samples were ready for cell acquisition and analysis by flow cytometry after a single washing step with PBS. Data were collected, on an 8-color flow cytometer FACSCanto II (BD Biosciences; RRID: SCR_018056), using BD FACSDiva (version 8.0.1 for Windows; RRID: SCR_001456) software and subsequently analyzed using FlowJo (version 10 for Windows, BD Biosciences; RRID: SCR_008520) software. A full representative gating strategy of the CD57^+^CXCR3^+^CD8^+^ T-cell subset is illustrated in Supplementary Fig. S1.

### Next-generation sequencing

DNA extraction was performed on BMMCs or peripheral blood mononuclear cells, before treatment initiation. In brief, 1 to 5 × 10^6^ cells were counted from each patient’s sample, and resuspended in PBS. The genomic DNA was isolated utilizing the PureLink Genomic DNA Mini Kit (Invitrogen, # K182001). Before next-generation sequencing, qualitative and quantitative evaluation of the isolated DNA samples was performed using NanoDrop 2000/2000c (Thermo Fisher Scientific; RRID: SCR_020309). The VARIANTPlex Myeloid panel (IDT) was utilized to detect copy number variations, single-nucleotide variants and indels in 75 myeloid associated genes as per manufacturer’s instructions. Target-enriched libraries of extracted nucleic acids for next-generation sequencing were prepared using anchored multiplex PCR, which leverages unidirectional gene-specific primers, sample indexes and molecular barcodes for multiplex targeted sequencing. Adapters ligated to the molecules before amplification carried molecular barcodes, enabling accurate unique molecule counting and error correction for robust mutation detection. Dual, independent coverage across all target regions ensured retention of some reads even if single-nucleotide variants blocked primer binding, enhancing reliability. The open-ended amplification provided flexibility for detecting novel RNA fusions and offers strand-specific bidirectional coverage in DNA. Sequencing was performed on an Illumina platform, and subsequent analysis was conducted using Archer (RRID: SCR_015854) Analysis bioinformatics platform.

### scRNA-seq and data processing

BMMCs were thawed and washed with RPMI-1640 (GlutaMAX, Gibco, # 61870) supplemented with 10% heat-inactivated FBS(Gibco, # 10270) and 100 U/mL penicillin–streptomycin (10,000 U/mL, Gibco, #15140). The BMMCs were treated with DNAse I 1 mg/mL (Sigma-Aldrich) for 10 minutes at room temperature. Samples were stained with 7-AAD Viability Staining Solution (420404, BioLegend), CD45 APC/Cy7 (304014, BioLegend; RRID: AB_314402), CD3 PE (317308, BioLegend; RRID: AB_571913), CD8 APC (345775, BD Biosciences; RRID: AB_2868803), and CD4 FITC (345768, BD Biosciences; RRID: AB_2868797). After antibody staining, cells were incubated with cell multiplexing oligos (3′ CellPlex Kit Set A, 10X Genomics) according to the manufacturer’s instructions. Based on the 7-AAD^−^CD3^+^CD4^−^CD8^+^ profile, cells were sorted using FACSAria III v8.0.1 software (BD Biosciences; RRID: SCR_016695). Flow sorting compensation was initially performed using single-stain controls and was further refined with small changes based on fluorescence minus one controls for each antibody. For the flow sorting process, a 70-μm nozzle was used, and the pressure was set at 70 psi. The electronic abort was close to zero to minimize cell loss. The cell purity was >95%.

Sorted cells were counted and resuspended in PBS + 10% FBS at a concentration of 1,600 cells/μL, and samples were combined by three per well of the Chromium Next GEM Chip G (10X Genomics) before being loaded onto the Chromium Controller (10X Genomics; RRID: SCR_019326). Pooling patient samples into groups of three per reaction ultimately resulted in three scRNA-seq libraries encompassing nine samples. Per pooled reaction, the discrimination of single cells and their allocation to each patient sample were performed with the assistance of cell multiplexing oligos. Samples were processed for single-cell encapsulation, cDNA, and cell multiplexing library generation using Chromium Next GEM Single Cell 3′ Reagent Kits v3.1 (Dual Index; 10X Genomics). The constructed libraries were sequenced on a NovaSeq 6000 sequencer (RRID: SCR_016387) with paired-end read sequencing. The 10X Genomics Cell Ranger (RRID: SCR_023672) multi v7.1.0 pipeline was used to map the sequencing reads to the human genome (GRCh38) and generate gene expression and feature barcode matrices. We specified r1-length and r2-length to 28 + 90, respectively, for both gene expression and feature libraries while preserving all other parameters of the pipeline under default settings. Supplementary Figure S2A depicts the GEX Barcode Rank Plots for each library as generated in the web summary output file of the CellRanger multi. Additionally, the results of the web summaries suggested that most of the sequencing reads were assigned to cell-associated barcodes (Supplementary Fig. S2B), as shown under the “Metrics Per Physical Library” section. Ideally, more than 70% of the sequencing reads should be mapped to cell-associated barcodes, and significantly lower values than this threshold would be strong indications for the need to perform ambient RNA correction according to 10X Genomics. As this particular metric was significantly higher than the ideal threshold value given by 10X Genomics, we concluded that estimation and correction for ambient RNA contamination was not critical for these libraries. The generated matrices consisted of 38,298 cellular barcodes spanning all samples, with a sequencing depth of 34,822 mean reads per barcode (cDNA) and were inserted into the R (Version 4.1.1) software package Seurat (v4.3.0; RRID: SCR_016341; ref. 27) for all downstream analyses. The gene expression matrices were filtered to discard cells expressing less than 200 genes and genes found in less than three cells. scRNA-seq data was processed in R using the Seurat package. Each library underwent independent sample demultiplexing and singlet identification using Seurat’s HTODemux function, which was applied to the CellRanger-generated feature barcode matrices. To illustrate the demultiplexing workflow, Supplementary Fig. S3 shows dot plots of cell multiplexing oligo expression patterns across all detected cell barcodes in library 3. The Seurat demultiplexing pipeline identified and excluded inter-sample doublets, retaining only sample-specific singlets. These filtered singlets were then merged into a unified Seurat object. Quality control metrics from the standard Seurat analysis pipeline are presented in Supplementary Fig. S4A and S4B, including violin and feature plots displaying: (1) mitochondrial gene percentage, (2) total RNA counts (nCount_RNA), and (3) number of detected genes (nFeature_RNA) per cell. These metrics are shown both for the initial singlet population following “HTODemux” analysis and for the final filtered dataset. During quality control of the combined dataset, cells expressing less than 200 or more than 4,500 genes and having more than 13% of mitochondrial associated genes were removed from further analysis. In addition, we have used the nFeature_RNA parameter to exclude cells with high probability of being intra-sample doublets because of the high number of uniquely expressed genes. Overall, doublet detection was performed in two steps considering the expression profiles of the cell multiplexing oligos and quality control metrics.

Gene expression data of the remaining 28,585 cells that passed quality control were normalized and scaled using the “LogNormalize” method and “ScaleData” command, respectively, whereas variable features were identified using the “FindVariableFeatures” command. Algorithm Harmony (RRID: SCR_022206; ref. 28) was used to perform batch correction and for further clustering of the data. Distribution of cells composing the final dataset in two-dimensional space pre- and postharmony integration and their UMAP visualization using PCA or harmony dimensionality reduction as input are displayed in Supplementary Fig. S5A and S5B. The first 63 principal components of the harmony reduction were selected based on the Seurat Elbow plot and were designated for the “dims” argument of the “FindNeighbors” and “RunUMAP” functions. A resolution of 0.3 was selected for graph-based cluster identification in the “FindClusters” function. Finally, cluster visualization in a two-dimensional space was performed using nonlinear dimensional reduction via UMAP. The “FindAllMarkers” command was implemented to identify cluster-defining genes expressed at least in 20% of the cluster cells at a minimum of a 0.25-log-fold difference between the respective cluster and the residual cells in the dataset. Contaminating clusters comprising non-CD8^+^ T cells were identified via gene expression profiles and were removed from subsequent analysis. To identify differentially expressed markers between conditions in the same cluster(s), the “FindMarkers” command with the MAST (RRID: SCR_016340) statistical test was used. For increased sensitivity, the output of the “FindMarkers” comparison contained genes with an adjusted *P* value <0.05, expressed in at least 5% of the cells under at least one condition, without implementing log-fold change thresholds. Calculation of curated gene set scores were calculated using the AUCell (RRID: SCR_021327) package (29). Enrichment pathway analysis of the differentially expressed genes was performed using the Enrichr (RRID: SCR_001575) tool (30, 31).

### Gene regulatory network analysis

The single-cell RNA-seq data underwent further bioinformatic analysis through the utilization of SCENIC (v1.3.1; RRID: SCR_017247). SCENIC is a computational tool that constructs intricate gene regulatory networks and uncovers distinct cellular states within the framework of single-cell RNA-seq data (29). SCENIC relies on three main R/Bioconductor packages: GENIE3 (v1.20.0; RRID: SCR_000217), RcisTarget (v1.18.2; RRID: SCR_024860), and AUCell (v1.20.2; RRID: SCR_021327). GENIE3 (32) identifies potential targets of Transcription Factors (TF) by elucidating the coexpression relationships existing between these TFs and their corresponding targets. Subsequently, RcisTarget (29) is utilized to pinpoint direct targets through the analysis of cis-regulatory motifs and construct TF regulons. SCENIC’s AUCell algorithm (29) was used to quantify regulon activity in individual cells. This analysis encompassed three key steps: regulon identification, TF activity scoring, and enrichment analysis. We then performed differential regulon activity analysis across the previously established Seurat clusters. The results were visualized through multiple approaches: UMAPs to show regulon activity distribution, heatmaps displaying cluster-specific regulon states (based on AUCell-generated binary on/off values), and pie charts representing regulon prevalence. Our implementation followed the established SCENIC protocols from Aibar and colleagues (29), with additional methodological refinements based on Zhu and colleagues (33).

### Statistical analysis

A two-tailed unpaired Student *t* test or the Mann–Whitney U test was used to compare two groups, as appropriate. For the comparison of multiple groups, one-way ANOVA followed by a “two-stage” Benjamini, Krieger, and Yekutieli multiple comparison test or Kruskal–Wallis test followed by a “two-stage” Benjamini, Krieger, and Yekutieli multiple comparison test were used as appropriate. The independence between variables was assessed using the *χ*^2^ test and Fisher’s exact test. Kaplan–Meier analysis was used for survival analysis, and the log-rank test was used to assess significance. To explore the association between BM CD57^+^CXCR3^+^CD8^+^ T cells and response to treatment, univariate analysis was performed. The optimal CD57^+^CXCR3^+^ cutoff was determined by transformation of continuous variable to binary one through optimal scaling; in detail, discretization to seven groups, regularization using ridge regression, and 10-fold cross-validation were performed through SPSS CATREG procedure. To explore the independent correlations between BM CD57^+^CXCR3^+^CD8^+^ T cells along with mutational status, as well as age, gender, and IPSS-R (both score and components), with response to treatment, single univariate and separate multivariate analyses for each mutation of interest were performed using Cox proportional hazards regression analysis; every parameter that was significantly correlated in a certain univariate analysis (*P* ≤ 0.05) was treated as a potential independent parameter in the relevant multivariate analysis. Statistical analysis was performed using GraphPad prism 9 (version 9.0.0 for Windows, GraphPad Software, Inc.; RRID: SCR_002798), IBM SPSS Statistics software (version 26.0 for Windows, IBM Corporation; RRID: SCR_016479), and the Cytobank platform. The level of significance was established at *P* < 0.05.

### Ethics

This study was approved by the local ethics committee (reference number: 877). All patients provided written informed consent in accordance with the principles outlined in the Declaration of Helsinki.

### Data availability

The authors state that all data supporting this study are available in the main text or Supplementary Materials. The raw scRNA-seq data for this study have been deposited in the NCBI Gene Expression Omnibus repository (RRID: SCR_005012) and are accessible through accession number GSE250077.

In addition, all flow and mass cytometry data have been deposited and are available in the Zenodo (RRID: SCR_004129) repository (https://doi.org/10.5281/zenodo.13949296). All other raw data were provided by the corresponding authors upon request.

## Results

### Immunophenotypic analysis of BM immune cells from patients with myeloid neoplasms

**Figure 1:**
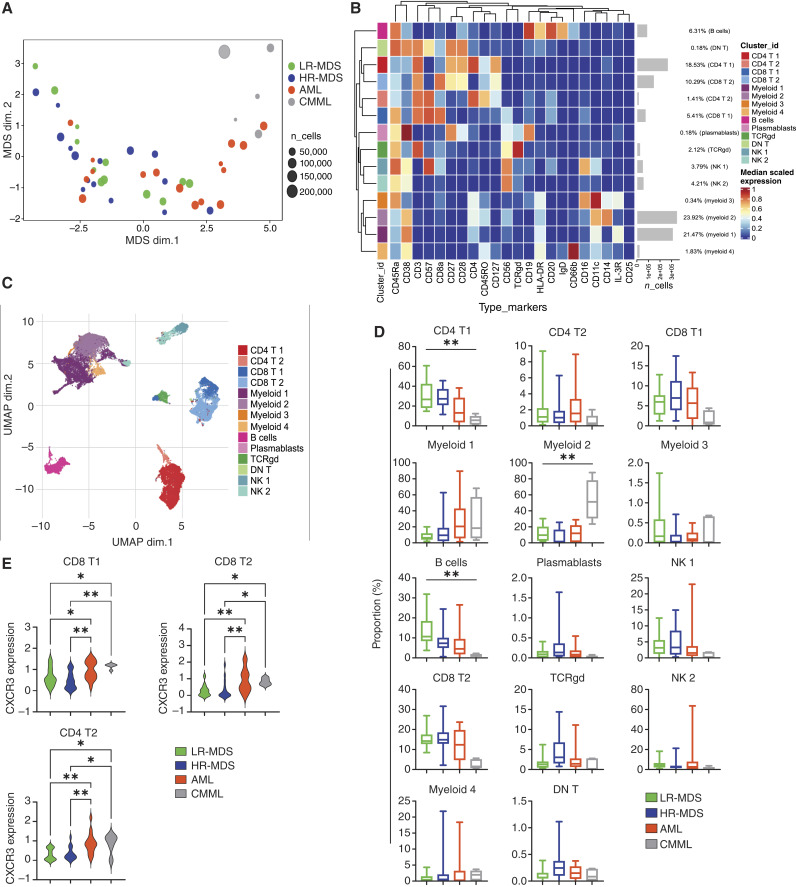
Untargeted analysis of CD45^+^ immune cells in patients with MDS, AML, and CMML by CyTOF. **A,** Multidimensional scale plot depicting the relationship between BM samples of patients with LR-MDS (*n* = 12), HR-MDS (*n* = 15), AML (*n* = 16), and CMML (*n* = 5). **B,** Heatmap showing the expression of the markers used for the characterization of each cell cluster. **C,** UMAP displaying the major immune cell clusters. **D,** Box charts displaying the frequency of each cell cluster. **E,** Violin plots showing the expression level of CXCR3 in the CD8 T1, CD8 T2, and CD4 T2 clusters, respectively. Kruskal–Wallis followed by the “two-stage” Benjamini, Krieger, and Yekutieli multiple comparison test was used in **D**. One-way ANOVA followed by the “two-stage” Benjamini, Krieger, and Yekutieli multiple comparison test was used in **E**. *, *P* < 0.05; **, *P* < 0.01.

To study the immune cell compartment, we performed deep immunophenotyping using CyTOF in BM samples from patients with LR-MDS (*n* = 12), HR-MDS (*n* = 15), AML (*n* = 16), and CMML (*n* = 5) collected before treatment initiation. Detailed demographic and clinical data are presented in Supplementary Table S1. Multidimensional scaling analysis was initially performed in samples derived from patients with MDS, AML and CMML (Fig. 1A). Untargeted cluster analysis identified 14 cell clusters (Fig. 1B and C). We observed a significantly decreased frequency of cells in the cluster of CD4^+^ T cells (CD4 T1) and B cells in patients with CMML, with a corresponding increase in the frequency of myeloid cluster 2 monocytic cells, characterized by the expression of CD11c, CD14, and CD38 (Fig. 1D). As chemokine signatures and the expression patterns of chemokine receptors potentially have prognostic implications in MDS and AML (34, 35), we further evaluated the expression of the chemokine receptors C–C chemokine receptor 4 (CCR4), CCR6, CCR7 and C–X–C Motif Chemokine Receptor 3 (CXCR3), CD161, and CD294 within the T cell clusters (Supplementary Fig. S6). We observed increased CXCR3 expression in cells from patients with AML and CMML compared with patients with MDS in the CD4 T2, CD8 T1, and CD8 T2 clusters (Fig. 1E).

**Figure 2:**
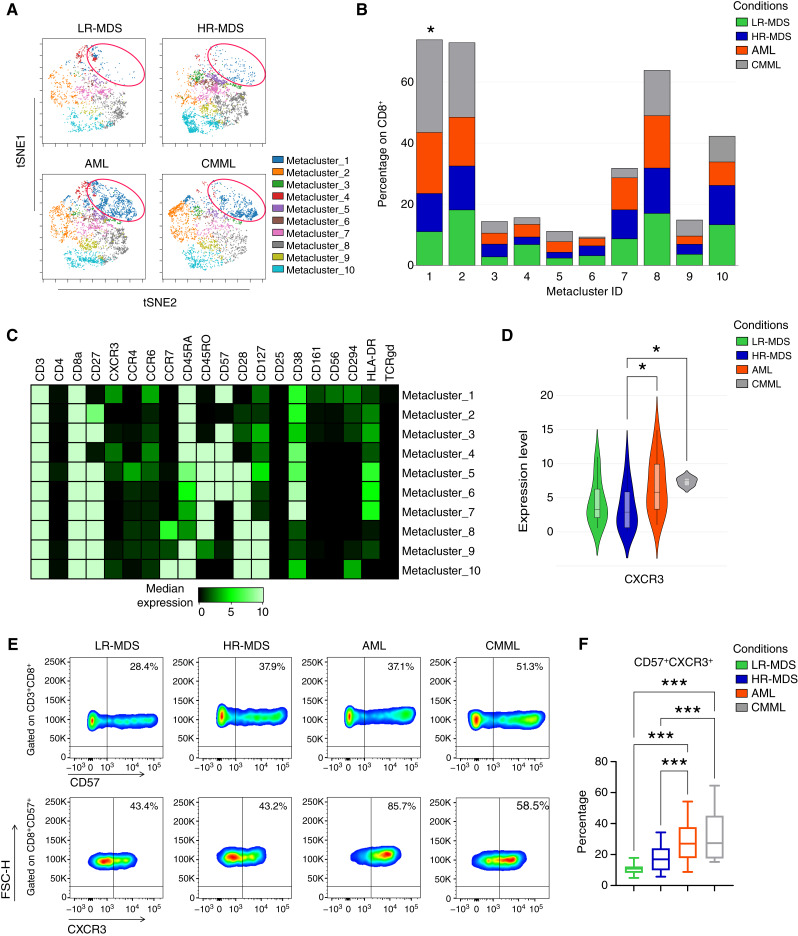
Identification of a CD8^+^ subpopulation (CD57^+^CXCR3^+^) which distinguishes patients with MDS from patients with AML and CMML. **A,** Representative viSNE plots, derived from the FlowSOM analysis of BM CD8^+^ T cells from patients with LR-MDS (*n* = 12), HR-MDS (*n* = 15), AML (*n* = 16), and CMML (*n* = 5). **B,** Bar plots displaying the proportion of the metaclusters between the groups, expressed as percentage within CD8^+^ T cells. **C,** Heatmap depicting the expression level of the T-related markers between the metaclusters. **D,** Violin plots showing the expression level of CXCR3 in metacluster 1. **E,** Representative flow cytometry plots for the identification of the CD57^+^CXCR3^+^CD8^+^ T cell subpopulation in a cohort of patients with LR-MDS (*n* = 7), HR-MDS (*n* = 27), AML (*n* = 20), and CMML (*n* = 10). **F,** Percentage of CD57^+^CXCR3^+^ cells within CD8^+^ T cells. Kruskal–Wallis was used in **B** and **D**. One-way ANOVA followed by the “two-stage” Benjamini, Krieger, and Yekutieli multiple comparison test was used in **F**. *, *P* < 0.05; ***, *P* < 0.001.

Based on these findings, we focused on CD4^+^ and CD8^+^ T cells and performed an untargeted cluster analysis of CD3^+^CD4^−^CD8^+^ (Fig. 2) and CD3^+^CD4^+^CD8^−^ T cells (Supplementary Fig. S7A–S7C). Ten clusters of CD8^+^ T cells were identified (Fig. 2A). We noted a significantly decreased frequency of cells within cluster 1 in samples from patients with LR-MDS and HR-MDS compared with AML and CMML (Fig. 2B), a cluster that included terminal effector cells (CCR7^−^CD45RA^+^) expressing CD57 and CXCR3 (Fig. 2C). We also identified an additional cluster (cluster 2) of terminal effector cells (CCR7^−^CD45RA^+^) that did not express CD57 and CXCR3 and did not show any differences among the groups (Fig. 2B and C). Additionally, the expression of CXCR3 in cells from cluster 1 was higher in patients with AML and CMML than in those with MDS (Fig. 2D). A similar analysis of CD4^+^ T cells did not reveal any differences in the frequency of generated clusters (Supplementary Fig. S7A–S7C).

To further confirm the above findings, we performed conventional flow cytometry on BM cells from an additional cohort of patients (*n* = 64), focusing on the frequency of CD57^+^CXCR3^+^CD8^+^ T cells (Fig. 2E and F). We also analyzed with flow cytometry samples from eight patients that were also analyzed with CyTOF, to compare the two methods. We observed that the frequency of this cell population within CD8^+^ T cells was higher in patients with AML and CMML than in those with LR-MDS and HR-MDS (Fig. 2F), which is consistent with the findings from the unsupervised analysis of data derived from CyTOF. In contrast, no difference was observed in the frequency of CD57^+^CXCR3^−^ CD8^+^ T cells in Supplementary Fig. S8.

Next, we characterized CD57^+^CXCR3^+^ CD8^+^ T cells compared with CD57^+^CXCR3^−^ CD8^+^ T cells using mass cytometry data. Increased expression of chemokine receptors CCR4 and CCR6 and decreased expression of the co-stimulatory molecules CD27 and CD28 were observed in CXCR3^+^ cells from patients with HR-MDS and AML (Supplementary Fig. S9A and S9B). Additionally, flow cytometry analysis revealed decreased PD-1 expression in CXCR3^+^ cells compared with that in CXCR3^−^ cells (Supplementary Fig. S10).

### The frequency of CD57^+^CXCR3^+^CD8^+^ T cells is associated with the response to AZA

**Figure 3:**
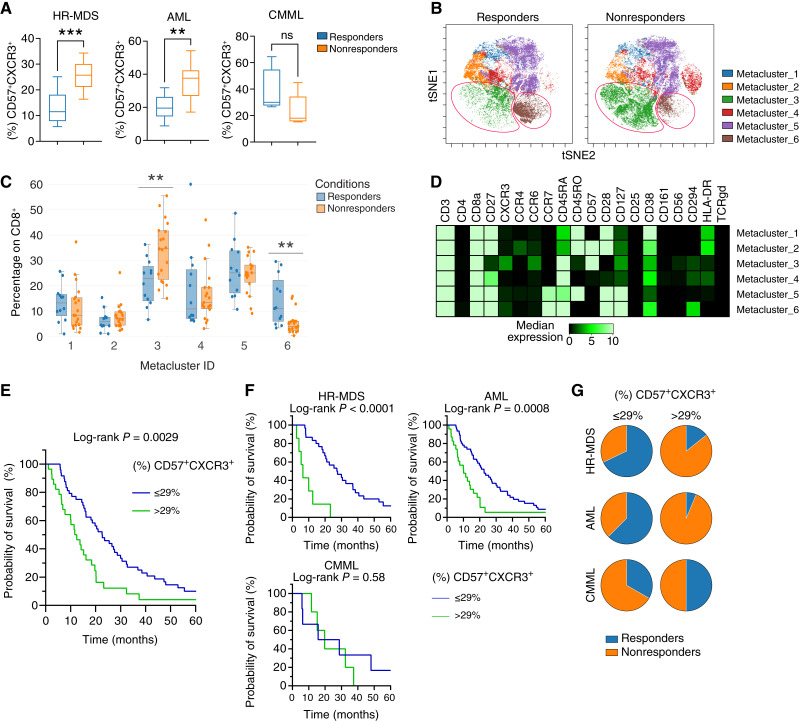
Association between the frequency of CD57^+^CXCR3^+^CD8^+^ T cells and outcome in patients with HR-MDS and AML under treatment with AZA. **A,** Box plots displaying the percentage of the CD57^+^CXCR3^+^ cells within CD8^+^ T cells, assessed by flow cytometry in responders and nonresponders (HR-MDS, *n* = 12 responders and 9 nonresponders; AML, *n* = 9 responders and 11 nonresponders; CMML, *n* = 5 responders and 5 nonresponders). **B,** After stratification of patients with HR-MDS and AML to responders (*n* = 12) and nonresponders (*n* = 19), FlowSOM analysis was performed on BM CD8^+^ T cells, which generated six metaclusters that are projected onto the viSNE plots. Representative viSNE plots (one for each group) are shown. **C,** Box plots showing the proportion of all metaclusters, expressed as the frequency within CD8^+^ T cells. **D,** Heatmap depicting the expression levels of all T-related markers. **E,** Kaplan–Meier curves for OS in patients which received AZA treatment, with ≤29% (*n* = 51) and >29% (*n* = 26) CD57^+^CXCR3^+^ CD8^+^ T cells before treatment initiation. The survival curves were compared by the log-rank (Mantel–Cox) test, and the *P* value is shown. The median OS of the ≤29% group was 20.98 months, whereas the median OS of the >29% group was 12.05 months. **F,** Survival curves for each disease subgroup. Increased (%) CD57^+^CXCR3^+^ correlates significantly with worse survival in patients with HR-MDS and AML, whereas no association is observed in patients with CMML. **G,** Patients with HR-MDS and AML with ≤29% CD57^+^CXCR3^+^ exhibited higher response rates. No association between the frequency of CD57^+^CXCR3^+^CD8^+^ T cells and response to therapy was observed in patients with CMML. An unpaired Student *t* test was used in **A**. A Mann–Whitney U test was used in **D**. **, *P* < 0.01; ***, *P* < 0.001.

Dynamic changes in chemokine receptor expression on T cells may indicate prognosis (35), whereas AZA may alter the BM chemokine profile in patients with MDS. Having observed a gradual increase in the frequency of the CD57^+^CXCR3^+^CD8^+^ T-cell subset from LR-MDS to AML, we next assessed whether the proportion of this cell population before AZA treatment initiation was associated with response to treatment. Univariate analysis revealed that a lower percentage of CD57^+^CXCR3^+^CD8^+^ T cells was associated with a better response (Supplementary Table S4). Furthermore, multivariate analysis confirmed the independent association between baseline frequencies of CD57^+^CXCR3^+^CD8^+^ T cells and patient responses (Supplementary Table S4). In line with this, using flow cytometry data, we observed that the baseline frequency of this cell subset was significantly increased in patients with HR-MDS and AML that did not respond to treatment, whereas no difference was observed in patients with CMML (Fig. 3A). Next, utilizing the CyTOF data, we performed unsupervised cluster analysis of CD3^+^CD4^−^CD8^+^ T cells in responders and nonresponders to AZA in patients with MDS and AML, which resulted in the identification of six cell clusters (Fig. 3B). We observed an increased frequency of cluster 3 in nonresponders (Fig. 3B and C), a cluster of CCR7^−^CD45RA^+^ cells characterized by the expression of CD57 and CXCR3 (Fig. 3D). Conversely, we observed an increased frequency of cluster 6 in responders, characterized by the naïve/memory markers CD28, CD27, CCR7, and CD127 (Fig. 3C and D), whereas there was no difference between responders and nonresponders in the analysis of total CD3^+^CD8^−^CD4^+^ T cells (Supplementary Fig. S11A–S11C).

We further evaluated whether the baseline frequency of the CD57^+^CXCR3^+^CD8^+^ T-cell subset is associated with survival. To do so, the optimal CD57^+^CXCR3^+^ cutoff value was determined to be >29% by transformation of the continuous variable to a binary variable through optimal scaling. We observed that patients with ≤29% CD57^+^CXCR3^+^CD8^+^ T cells had better overall survival (Fig. 3E). However, when patients with HR-MDS, AML, and CMML were evaluated separately, we observed that this cutoff value could be applied to patients with HR-MDS and AML but not to patients with CMML (Fig. 3F). We next assessed the response rate to AZA using this cutoff value. Patients with HR-MDS and AML, characterized by a baseline frequency of ≤29% CD57^+^CXCR3^+^CD8^+^ T cells, demonstrated higher rates of response to AZA (Fig. 3G). The frequency of CD57+CXCR3+CD8+ T cells was not correlated with treatment response in patients with CMML (Fig. 3G), which further illustrates the distinct immunologic profile of this disease. In patients with MDS and AML treated with AZA, elevated T-cell counts predicted both treatment failure and reduced survival.

### Association between mutational status and the frequency of CD57^+^CXCR3^+^CD8^+^ T cells

We further investigated whether there was a correlation between mutational burden, type of mutation, and frequency of CD57^+^CXCR3^+^CD8^+^ T cells in patients with HR-MDS and AML. No difference was found in the frequency of this cell population when the patients were categorized based on oncogenic mutations (Supplementary Fig. S12A), whereas no single mutation was associated with an increased frequency (>29%) of the CD57^+^CXCR3^+^CD8^+^ T-cell subset (Supplementary Fig. S12B). Furthermore, the number of oncogenic mutations was not significantly altered in patients with >29% CD57^+^CXCR3^+^CD8^+^ T cells compared with that in patients with a lower frequency (≤29%; Supplementary Fig. S12C), and no specific group of oncogenic mutations was associated with the frequency of CD57^+^CXCR3^+^CD8^+^ T cells (Supplementary Fig. S12D).

### Single-cell transcriptomic landscape of CD8^+^ T cells in patients with HR-MDS and secondary AML

**Figure 4:**
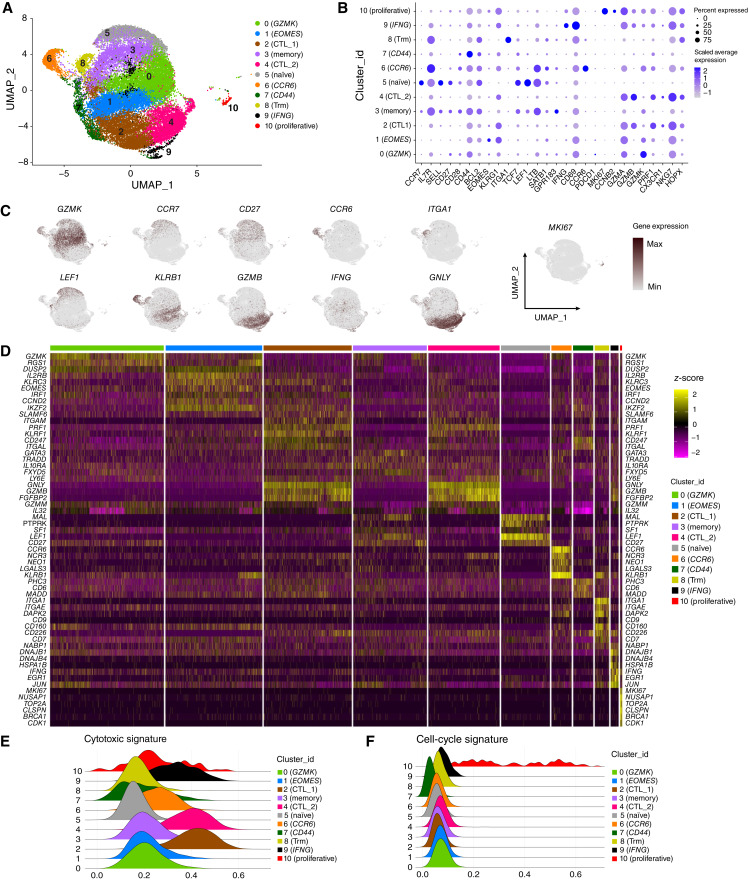
Profiling of BM-derived CD8^+^ T cells of patients with HR-MDS and secondary AML with scRNA-seq. **A,** UMAP of CD8^+^ T cells identified 11 clusters. A total of 28,449 CD8^+^ T cells were pooled from four patients with HR-MDS (15,597 cells) and five patients with secondary AML (12,852 cells). **B,** Bubble plot depicting the average expression of genes used to characterize the clusters. **C,** Expression of selected genes projected onto UMAPs. **D,** Heatmap showing selected top DEGs for each cell cluster. **E,** Ridgeline plots displaying the cytotoxic signature score for each cell cluster, as defined by the expression of key-related genes. **F,** Ridgeline plots displaying the cell-cycle signature score for each cell cluster.

We next sought to assess the molecular signature of BM CD8^+^ T cells from patients with MDS and AML secondary to MDS at the single-cell level to further identify the molecular signatures in specific CD8^+^ T-cell subpopulations associated with disease progression and clinical outcomes of AZA monotherapy. scRNA-seq was performed on sorted BM CD8^+^ T cells from patients with HR-MDS (*n* = 4) and secondary AML (*n* = 5), before AZA initiation (Supplementary Table S5). We obtained transcriptomes of 28,449 cells in total. Based on unsupervised clustering, cells were partitioned into 11 clusters (Fig. 4A), which were characterized according to the gene expression of markers associated with T-cell phenotype (36), including naïve/memory markers (*CCR7*, *IL7R*, *SELL*, *CD27*, *CD28*, and *CD44*), cytotoxic markers (*GZMA*, *GZMB*, *GZMK*, *PRF1*, *CX3CR1*, *NKG7*, *HOPX*, and *KLRG1*), cell cycle genes (*MKI67* and *CCNB2*), TFs (*LEF1*, *EOMES*, and *TCF7*), the cytokine *IFNG*, and the cell surface markers *ITGA1*, *CD69*, and *CCR6* (Fig. 4B and C). Differential expression data are reported in Supplementary Data Sheet S1. We identified clusters of cells characterized by the expression of *GZMK* (cluster 0) and *EOMES*, *KLRG1*, and *GZMK* (cluster 1), previously characterized as predysfunctional cells in studies of patients with solid tumors (36, 37). We further identified two clusters of cytotoxic CD8^+^ T lymphocytes, clusters 2 (CTL_1) and 4 (CTL_2), based on the expression of *GZMA*, *GZMB*, and *PRF1* (38). Clusters 1, 2, and 4 had the highest frequency of cells expressing *B3GAT1*, a gene encoding CD57 (Supplementary Fig. S13). We identified a cluster of memory-like cells characterized by the expression of *IL7R* and low expression of *CCR7*, *SELL*, and *CD27* (cluster 3), a cluster of naïve-like cells that expressed *CCR7*, *SELL*, and *CD27* and the TFs *TCF7* and *LEF1* (cluster 5; ref. 39), a cluster characterized by the expression of *CCR6* (cluster 6), and a cluster characterized by the highest expression of *CD44* (cluster 7; Fig. 4B and C). Cluster 8 included cells expressing *ITGA1* and *ITGAE* (Fig. 4D), previously described as resident memory cells (cluster 8; refs. 39–41), and cluster 9 included cytotoxic cells expressing *IFNG*, *GZMK*, and *NKG7*. Finally, a cluster of proliferating T cells (Cluster 10) was identified (Fig. 4B and C). The top differentially expressed genes (DEG) for each cell cluster are shown in Fig. 4D. We further used a previously reported cytotoxic score (42) to confirm the enhanced cytotoxicity of cells in clusters 2 (CTL_1), 4 (CTL_2), and 9 (*IFNG*; Fig. 4E), and a cell cycle score (42) to confirm the increased proliferation activity of cells in cluster 10 (proliferative; Fig. 4F). Finally, utilizing a previously reported dysfunctional/exhaustion score (42) we did not identify a specific cluster characterized by the high expression of genes associated with exhaustion, such as *PDCD1*, *LAG3*, *HAVCR2*, *ENTPD1*, or *CTLA4* (Supplementary Fig. S14A; ref. 36). Nevertheless, cluster 1 (*EOMES*) exhibited the highest dysfunctional score among clusters with the highest cell abundance (Supplementary Fig. S14B).

We further investigated whether there was a difference in the transcriptomic landscape of CD8^+^ T cells between HR-MDS and secondary AML (Supplementary Fig. S15A). Differential expression data are reported in Supplementary Data Sheet S2. We observed that the frequency of cells in cluster 4 (CTL_2) was increased in patients with AML (Supplementary Fig. S15B and S15C), a cluster showing high expression levels of *B3GAT1*, which encodes the CD57 protein (Supplementary Fig. S13). Pathway analysis of the DEGs in cluster 4 (CTL_2; Supplementary Fig. S15D) revealed an overrepresentation of the T-cell receptor pathway and anabolic pathways involved in DNA transcription, gene expression, mRNA processing, and cell cycle in cells from patients with HR-MDS, and IFN response pathways and the oxidative phosphorylation pathway in cells from patients with AML (Supplementary Fig. S15E). Notably, when we studied the exhaustion signature (42) between the two groups in cluster 1 (*EOMES*), a cluster characterized by the highest dysfunctional score among the clusters, we observed a significantly higher dysfunctional score in the HR-MDS group (Supplementary Fig. S15F), which was associated with enhanced expression of *CD7*, *FAM3C*, *TIGIT*, *TNFRSF9*, *DGKH*, *LYST*, *RAB27A*, *TNFRSF1B* (Supplementary Fig. S15G) and *PDCD1*, *CD244*, *AKAP5*, and *KIR2DL4* (Supplementary Fig. S16). Taken together, CD8^+^ T cells in AML have a distinct molecular profile compared with MDS, exhibiting an increased frequency of CTLs with an IFN-related signature.

### Single-cell transcriptomes of CD8^+^ T cells are associated with treatment outcome

**Figure 5:**
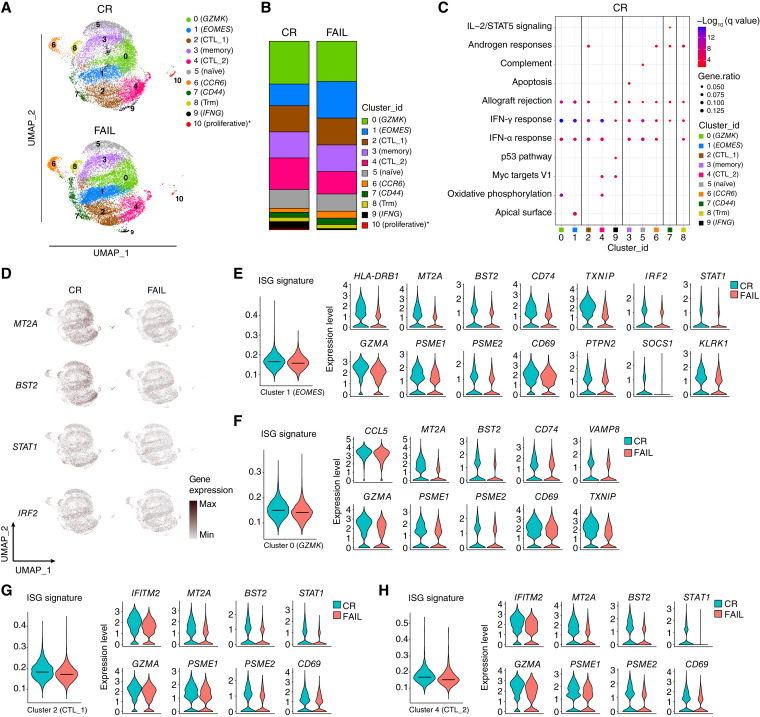
BM-derived CD8^+^ T cells from responders (CR) to AZA show an enhanced ISG molecular signature compared with nonresponders (FAIL) in scRNA-seq analysis. **A,** Comparison of separate UMAPs for CR (a total of 13,667 cells, including 7,571 from patients with MDS and 6,096 cells from patients with secondary AML, respectively) and FAIL patients (a total of 14,782 cells, including 8,026 cells from patients with MDS and 6,756 cells from patients with secondary AML, respectively). **B,** Stacked bar chart showing the average distribution of clusters between the two groups. The percentage of cluster 10 (proliferative) was increased in CR compared with FAIL patients (unpaired Student *t* test, *P* = 0.0418). **C,** Dot plot representing MSigDB (Hallmark 2020) enrichment analysis of positively enriched pathways in CR patients. Enriched pathways with a *q*-value <0.05 (Benjamini–Hochberg correction) are shown. **D,** Gradient expression of representative selected genes involved in IFN-related pathways, as they are projected onto UMAPs. **E,** ISG score of cluster 0 (*GZMK*) and violin plots showing the expression levels of the top differentially expressed IFN-stimulated genes of cluster 0 (*GZMK*) between CR and FAIL. **F,** ISG score of cluster 1 (*EOMES*) and violin plots showing the expression levels of the top differentially expressed IFN-stimulated genes of cluster 1 (*EOMES*) between CR and FAIL. **G,** ISG score of cluster 2 (CTL_1) and violin plots displaying the expression levels of the top differentially expressed IFN-stimulated genes of cluster 2 (CTL_1) between CR and FAIL. **H,** ISG score of cluster 4 (CTL_2) and violin plots displaying the expression levels of the top differentially expressed IFN-related genes of cluster 4 (CTL_2) between CR and FAIL.

We next sought to identify the molecular signatures in CD8^+^ T-cell subpopulations associated with AZA responses (Fig. 5A). We did not observe any statistically significant difference in the cluster abundance in patients who achieved complete remission (CR) after treatment with AZA (*n* = 2 patients with HR-MDS, 7,571 cells; *n* = 2 patients with secondary AML, 6,096 cells) and patients who failed treatment (FAIL; *n* = 2 patients with HR-MDS, 8,026 cells; *n* = 3 patients with secondary AML, 6,756 cells; Fig. 5B), except for an increased abundance of cluster 10 (proliferative) in the CR group (Supplementary Fig. S17). Differential expression data are reported in Supplementary Data Sheet S3.

Pathway analysis revealed that the DEGs upregulated in the CR group were associated with the IFN response in all clusters (Fig. 5C). Increased expression of the IFN-responsive genes *MT2A*, *BST2*, *STAT1*, and *IRF2* was observed in the CR group (Fig. 5D). We then focused on specific clusters and used a score derived from the expression of IFN-stimulated genes (ISG signature; ref. 43). We observed higher ISG signature scores in the CR group in cluster 0 (*GZMK*), which was associated with increased expression of the IFN-responsive genes *CCL5*, *MT2A*, *BST2*, *CD74*, *GZMA*, *PSME1*, *PSME2*, *CD69*, *VAMP8*, and *TXNIP* (Fig. 5E). Similarly, increased expression of *HLA-DRB1*, *MT2A*, *BST2*, *CD74*, *TXNIP*, *GZMA*, *PSME1*, *PSME2*, *CD69*, *PTPN2*, *IRF2*, *STAT1*, *SOCS1*, and *KLRK1* was observed in cluster 1 (*EOMES*; Fig. 5F). With regard to the cytotoxic clusters 2 (CTL_1; Fig. 5G; Supplementary Fig. S18A) and 4 (CTL_2; Fig. 5H; Supplementary Fig. S18B), a higher ISG signature and increased expression of *IFITM2*, *MT2A*, *BST2*, *STAT1*, *GZMA*, *PSME1*, *PSME2*, and *CD69* were detected in cells from the CR group from both clusters.

**Figure 6:**
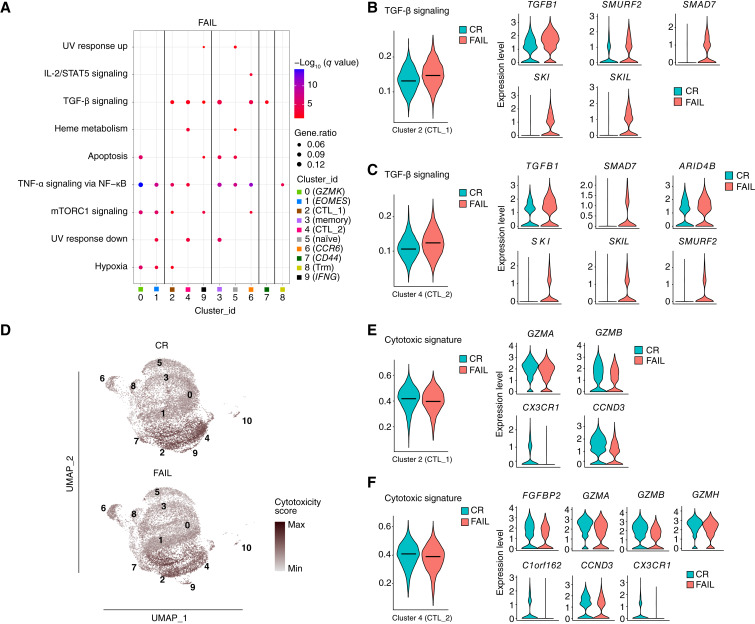
BM-derived CD8^+^ T cells of nonresponders (FAIL) displayed a suppressed cytotoxic molecular signature at the single-cell level. **A,** Dot plot representing MSigDB (Hallmark 2020) enrichment analysis of positively enriched pathways in FAIL patients. Enriched pathways with a *q*-value <0.05 (Benjamini–Hochberg correction) are shown. **B,** TGF-β signaling score of cluster 2 (CTL_1) and violin plots displaying the expression levels of the top DEGs of cluster 2 (CTL_1), involved in the enrichment of the TGF-β signaling pathway between CR and FAIL. **C,** TGF-β signaling score of cluster 4 (CTL_2) and violin plots displaying the expression levels of the top DEGs of cluster 4 (CTL_2), involved in the enrichment of the TGF-β signaling pathway between CR and FAIL. **D,** Comparison of the cytotoxic score of each group, as it is projected onto the respective UMAPs. **E,** Cytotoxic score of cluster 2 (CTL_1) and violin plots exhibiting the expression levels of the top differentially expressed cytotoxicity-related genes of cluster 2 (CTL_1) between CR and FAIL. **F,** Cytotoxic score of cluster 4 (CTL_2) and violin plots exhibiting the expression levels of the top differentially expressed cytotoxicity-related genes of cluster 4 (CTL_2) between CR and FAIL.

Pathway analysis of DEGs upregulated in the FAIL group demonstrated that cells from clusters 0 (*GZMK*), 1 (*EOMES*), 2 (CTL_1), 3 (memory), 4 (CTL_2), 5 (naive), 6 (*CCR6*), and 8 (Trm) were enriched for DEGs associated with TNF signaling (Fig. 6A; Supplementary Fig. S19A–S19D). Moreover, cells from cytotoxic clusters 2 (CTL_1), 4 (CTL_2), and 9 (*IFNG*) and clusters 3 (Memory), 6 (*CCR6*), and 7 (*CD44*) were enriched for DEGs involved in the TGF-β signaling pathway (Fig. 6A). Specifically, for cluster 2 (CTL_1), there was a higher TGF-β signature score in the FAIL group and an increased expression of *TFGB1*, *SMURF2*, *SMAD7*, *SKI*, and *SKIL* (Fig. 6B; Supplementary Fig. S18A), whereas for cluster 4 (CTL_2), a higher TGF-β signature score was associated with an increased expression of *TFGB1*, *ARID4B*, *SMAD7*, *SKI*, *SKIL*, and *SMURF2* (Fig. 6C; Supplementary Fig. S18B). Because TGF-β signaling has been previously associated with decreased cytotoxicity (44) we used the cytotoxic signature score (Fig. 6D) to determine whether the TGF-β signature is associated with decreased cytotoxic activity in CTL clusters in the FAIL group. We observed higher cytotoxicity scores in the CR group in cluster 2 (CTL_1), which was associated with enhanced expression of *GZMA*, *GZMB*, *CX3CR1*, and *CCND3* (Fig. 6E), and in cluster 4 (CTL_2), which was associated with enhanced expression of *FGFBP2*, *GZMA*, *GZMB*, *GZMH*, *CCND3*, *C1orf162*, and *CX3CR1* (Fig. 6F). These data show that an IFN-related signature is associated with treatment response, whereas TGF-β signature is associated with treatment failure, which could be, at least in part, the result of decreased cytotoxic activity.

We further used the dysfunctional score (42) to investigate potential differences in the expression of exhaustion-associated genes according to treatment outcomes. This analysis revealed no significant differences in dysfunctional scores across all clusters between the CR and FAIL patients (Supplementary Fig. S20).

**Figure 7:**
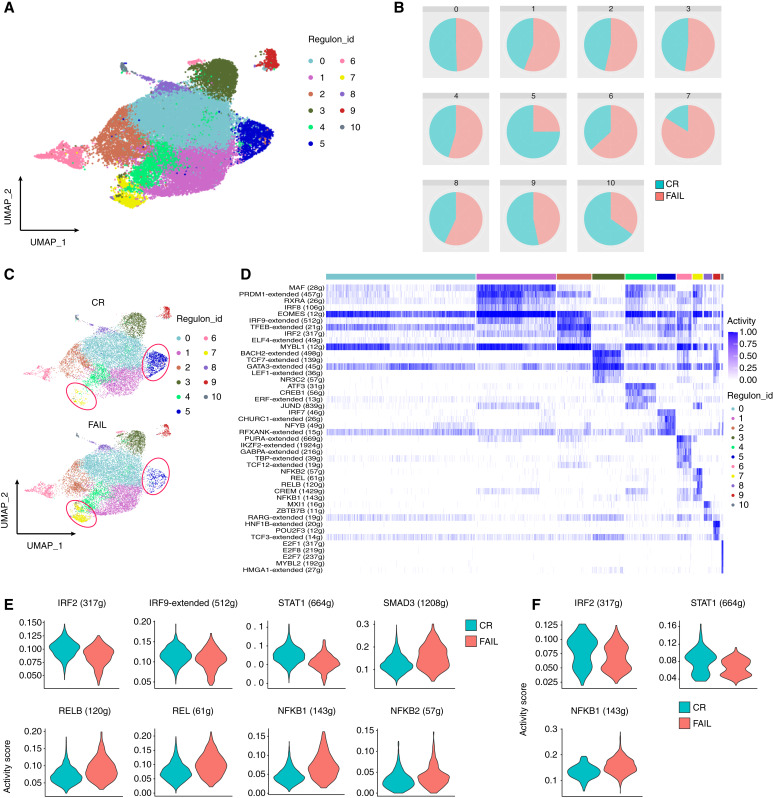
TF regulatory network analysis in BM-derived CD8^+^ T cells. **A,** UMAP depicting the clustering of CD8^+^ T cells based on regulons. **B,** Pie charts illustrating the representation of cells from CR and FAIL patients within each regulon. **C,** Comparison of cell distribution in regulons between the groups using separate UMAPs for each group. **D,** Heatmap showing the top differentially activated TFs of each regulon cluster. **E** and **F,** Violin plots depicting the activity score of selected TFs per sample type in regulons 5 and 7, respectively.

Cytokines interact with TFs to regulate T-cell fate and functionality (45). To identify TFs that could act as possible regulators of the transcriptomic alterations observed in responders to AZA compared with nonresponders, TF regulatory network analysis was performed using SCENIC (29), which resulted in the identification of 11 clusters-regulons (Fig. 7A). We observed that regulon 5 was enriched in cells from the CR group, whereas regulon 7 was enriched in cells from the FAIL group (Fig. 7B and C). Regulon cluster 5 was characterized by IRF7-, CHURC1-, NFYB-, and RFXANK-regulated networks (Fig. 7D) and was enriched mainly in cells from cluster 4 (CTL_2; Supplementary Fig. S21). On the other hand, regulon 7 was characterized by the NFKB2-, REL-, RELB-, CREM-, and NFKB1-regulated networks (Fig. 7D) and was enriched with cells from cluster 0 (*GZMK*) and cluster 1 (*EOMES*; Supplementary Fig. S21), which were previously described as predysfunctional cells (36, 37).

Differential TF activity analysis between the two groups of patients within regulon 5 predicted that the activity of the IFN-related TFs IRF2, IRF9, and STAT1 was increased in the CR group and the activity of SMAD3, and the NF-κB superfamily TFs RELB, REL, NFKB2, and NFKB1 was increased in the FAIL group (Fig. 7E). A similar analysis within regulon 7 predicted increased activity of IRF2 and STAT1 and decreased activity of NFKB1 in cells derived from the CR group (Fig. 7F). Taken together, TF regulatory network analysis further supports that BM CD8^+^ T cells in the CR group are targeted by TFs associated with IFN signaling, whereas TFs linked to TGF-β and NF-κB signaling target CD8^+^ T cells in the FAIL group.

## Discussion

Alterations in CD8^+^ T-cell functionality in the tumor milieu promote tumor evasion and compromise responses to immunotherapies (36). In AML and MDS, several CD8^+^ T-cell defects have been described that are potentially reversible by various treatments, including AZA (4, 8, 46). However, the architecture of CD8^+^ T-cell immunity and its impact on the clinical behavior of AML and MDS are still incompletely understood, thus hampering the development of successful immunotherapeutic approaches.

In this study, we present a comprehensive single-cell analysis of CD8^+^ T cells isolated from the BM immune microenvironment of patients with clonal myeloid disorders. Our focus was on identifying distinct immunophenotypic and molecular signatures correlated with the outcomes of AZA treatment.

Deep immunophenotyping with CyTOF identified a BM CD57^+^CXCR3^+^CD8^+^ T-cell population with an increased frequency in patients with AML and CMML compared with MDS. We further observed that an increased pretreatment frequency of BM CD57^+^CXCR3^+^CD8^+^ T cells was associated with poor overall survival and response to AZA treatment in patients with HR-MDS and AML. Interestingly, no association between the frequency of this cell population and the disease course was observed in patients with CMML, supporting the notion that this disorder does not share common immune features with MDS and AML. Although the mutational profile may sculpt specific immune response patterns across heterogeneous tumors (47), no definite association between somatic mutations and defects in T-cell immunity has been shown in MDS (48). Consistent with this report and our previous observation, showing the absence of an association between alterations in CD4^+^ T cells and somatic mutations (18), we could not find any correlation between the levels of the aforementioned subpopulation and the mutational profile of patients.

The expression of CD57 in CD8^+^ T cells coupled with the absence of CD28 expression characterizes a senescent-like phenotype linked to chronic immune activation in several disorders (49). These CD57^+^CD28^−^CD8^+^ T cells are antigen-specific effector cells that have limited proliferation capacity because of their advanced differentiation stage (50). Previous studies have demonstrated increased levels of CD57^+^CD28^−^CD8^+^ T cells in patients with MDS and AML compared with those in healthy individuals (9, 51). CXCR3, on the other hand, is expressed on Th1 CD4^+^ T cells (52) and effector CD8^+^ T cells and is considered crucial for the recruitment of T cells to inflammatory sites (52, 53). The expression of CXCR3 on CD8^+^ T cells has been associated with changes in the equilibrium from memory to effector cell populations (54). Interestingly, recent studies using scRNA-seq in CD8^+^ T cells isolated from patients with cancer have described cells expressing *CXCR3* together with *GZMK* and *EOMES* as a predysfunctional cell population (37).

In line with our findings, it has been recently demonstrated that the accumulation of senescent-like CD8^+^CD57^+^ T cells is negatively associated with the response to chemotherapy and checkpoint blockade immunotherapy in patients with AML (55). By using *in vitro* studies, the authors further demonstrated that patient-derived senescent-like T cells were not able to sufficiently eliminate autologous AML blasts compared with their nonsenescent CD8^+^ T-cell counterparts, providing direct evidence for their limited antileukemic activity (55).

Based on our immunophenotyping findings, we performed scRNA-seq analysis to investigate the transcriptomic profile of BM CD8^+^ T cells in patients with HR-MDS and secondary AML. We observed that the abundance of cells within the CTL_2 cluster, characterized by higher expression compared with other clusters of *B3GAT1*, the gene that encodes the CD57 protein, was increased in patients with secondary AML compared with those with HR-MDS, which is consistent with the immunophenotypic findings. With regard to the response to treatment, a significant enrichment of the TGF-β signaling pathway was observed in the cytotoxic clusters (CTL_1 and CTL_2) of nonresponders. This pathway has previously been shown to directly inhibit the cytotoxic activity of CD8^+^ T cells, leading to compromised antitumor responses, tumor evasion, and poor outcomes (44). In line with these findings, nonresponders displayed a decreased cytotoxic signature within the same clusters. Notably, upregulated TGF-β signaling has also been reported in *ex vivo*–expanded BM mesenchymal cells from AZA-treated patients (56), potentially implying ubiquitous targeting of TGF-β signaling by AZA. Luspatercept, an inhibitor of TGF-β signaling, has been recently approved by the FDA for the treatment of transfusion-dependent LR-MDS patients either after failure of erythropoiesis-stimulating agents or at the first line (57). The effect of luspatercept on tumor immunity is still unknown; however, given the immunoregulatory role of TGF-β, it could be worthwhile considering the potential benefit of adding luspatercept to AZA refractory patients.

In contrast, responders exhibited a significant enrichment of pathways associated with IFN responses across many BM CD8^+^ T-cell clusters. IFNs play a crucial role in immune activation during antitumor responses (58, 59). Notably, evidence indicates that AZA exerts its effects in an IFN-dependent manner by activating type I and III IFN signaling in tumor cell lines (60, 61), as well as upregulating the expression of ISGs, all of which aid in the rejuvenation of antitumor immunity (60). This process is suggested to be mediated by AZA-induced expression of endogenous retroelements (ERE), a subfamily of transposable elements (TE), driving the elevation of intracellular dsRNA levels and the activation of IFN I or III pathway, leading to a viral mimicry state (60, 61). AZA has been shown to increase the representation of EREs in BM hematopoietic stem cells of patients with MDS and CMML compared with their baseline levels; however, the upregulation of total EREs could not predict treatment response (62). Alternatively, investigation of the gene profile of malignant cells in patients with MDS, CMML, and AML showed that response to AZA was associated with upregulation of a specific subset of evolutionary young TEs and enrichment of the type I IFN pathway (63). Therefore, our findings further reinforce the concept of an IFN-mediated mechanism of AZA in patients with myeloid neoplasms, potentially working in synergy with TE induction.

Overall, the results suggest that the interplay between TGF-β and IFN signaling in CTLs within the BM microenvironment govern their antitumor activity, thereby influencing treatment responses.

Immunotherapies based on immune checkpoint blockade (ICB) have revolutionized the field of oncology (64). Many studies employing scRNA-seq in solid tumors have demonstrated that CD8^+^ T cells show a distinct molecular signature, indicative of an exhausted phenotype, which is associated with disease progression and treatment resistance (42, 65), thereby providing evidence supporting the effectiveness of ICB treatment in these patients. In contrast, ICB therapy for myeloid malignancies, including MDS and AML, has yielded limited results, and the risk of serious adverse events remains (66). Therefore, identifying alternative pathways, such as TGF-β signaling, which can be targeted by immunotherapies in patients with myeloid neoplasms is of critical importance.

In conclusion, our mass cytometry-guided transcriptomic analysis of BM CD8^+^ T cells at the single-cell level reveals distinct abnormalities linked to AZA response in patients with AML and MDS. Notably, we identified TGF-β signaling in BM CD8^+^ T cells as a potential immune-mediated mechanism driving AZA resistance, highlighting the therapeutic potential of targeting the TGF-β pathway to prevent or overcome AZA refractoriness. Recent data suggest a T cell–mediated antileukemic activity of venetoclax (46) and a triple combination of AZA, venetoclax, and luspatercept may have the potential to induce a fully competent immune-mediated control of the leukemic clone in patients with AML and MDS.

## Supplementary Material

Supplementary Data Supplementary Figures and Tables

Data sheet 1 Data Sheet 1

Data sheet 2 Data sheet 2

Data sheet 3 Data sheet 3

## References

[1] Arber DA , Orazi A, Hasserjian RP, Borowitz MJ, Calvo KR, Kvasnicka H-M, . International consensus classification of myeloid neoplasms and acute leukemias: integrating morphologic, clinical, and genomic data. Blood 2022;140:1200–28. 35767897 10.1182/blood.2022015850 PMC9479031

[2] Ogawa S . Genetics of MDS. Blood 2019;133:1049–59. 30670442 10.1182/blood-2018-10-844621 PMC6587668

[3] Itzykson R , Fenaux P. Epigenetics of myelodysplastic syndromes. Leukemia 2014;28:497–506. 24247656 10.1038/leu.2013.343

[4] Rodriguez-Sevilla JJ , Colla S. T cell dysfunctions in myelodysplastic syndromes. Blood 2024;143:1329–43. 38237139 10.1182/blood.2023023166

[5] Balderman SR , Li AJ, Hoffman CM, Frisch BJ, Goodman AN, LaMere MW, . Targeting of the bone marrow microenvironment improves outcome in a murine model of myelodysplastic syndrome. Blood 2016;127:616–25. 26637787 10.1182/blood-2015-06-653113 PMC4742549

[6] Pronk E , Raaijmakers MHGP. The mesenchymal niche in MDS. Blood 2019;133:1031–8. 30670448 10.1182/blood-2018-10-844639

[7] Verma NK , Wong BHS, Poh ZS, Udayakumar A, Verma R, Goh RKJ, . Obstacles for T-lymphocytes in the tumour microenvironment: therapeutic challenges, advances and opportunities beyond immune checkpoint. EBioMedicine 2022;83:104216. 35986950 10.1016/j.ebiom.2022.104216 PMC9403334

[8] Knaus HA , Berglund S, Hackl H, Blackford AL, Zeidner JF, Montiel-Esparza R, . Signatures of CD8^+^ T cell dysfunction in AML patients and their reversibility with response to chemotherapy. JCI Insight 2018;3:120974. 30385732 10.1172/jci.insight.120974 PMC6238744

[9] Radpour R , Riether C, Simillion C, Höpner S, Bruggmann R, Ochsenbein AF. CD8^+^ T cells expand stem and progenitor cells in favorable but not adverse risk acute myeloid leukemia. Leukemia 2019;33:2379–92. 30877275 10.1038/s41375-019-0441-9

[10] Raskov H , Orhan A, Christensen JP, Gögenur I. Cytotoxic CD8^+^ T cells in cancer and cancer immunotherapy. Br J Cancer 2021;124:359–67. 32929195 10.1038/s41416-020-01048-4 PMC7853123

[11] Liu X , Hoft DF, Peng G. Senescent T cells within suppressive tumor microenvironments: emerging target for tumor immunotherapy. J Clin Invest 2020;130:1073–83. 32118585 10.1172/JCI133679 PMC7269563

[12] Platzbecker U , Fenaux P. Recent frustration and innovation in myelodysplastic syndrome. Haematologica 2016;101:891–3. 27478197 10.3324/haematol.2016.142836 PMC4967566

[13] Stomper J , Rotondo JC, Greve G, Lübbert M. Hypomethylating agents (HMA) for the treatment of acute myeloid leukemia and myelodysplastic syndromes: mechanisms of resistance and novel HMA-based therapies. Leukemia 2021;35:1873–89. 33958699 10.1038/s41375-021-01218-0 PMC8257497

[14] Pleyer L , Leisch M, Kourakli A, Padron E, Maciejewski JP, Xicoy Cirici B, . Outcomes of patients with chronic myelomonocytic leukaemia treated with non-curative therapies: a retrospective cohort study. Lancet Haematol 2021;8:e135–48. 33513373 10.1016/S2352-3026(20)30374-4

[15] DiNardo CD , Jonas BA, Pullarkat V, Thirman MJ, Garcia JS, Wei AH, . Azacitidine and venetoclax in previously untreated acute myeloid leukemia. N Engl J Med 2020;383:617–29. 32786187 10.1056/NEJMoa2012971

[16] Prébet T , Gore SD, Esterni B, Gardin C, Itzykson R, Thepot S, . Outcome of high-risk myelodysplastic syndrome after azacitidine treatment failure. J Clin Oncol 2011;29:3322–7. 21788559 10.1200/JCO.2011.35.8135 PMC4859209

[17] Zhao G , Wang Q, Li S, Wang X. Resistance to hypomethylating agents in myelodysplastic syndrome and acute myeloid leukemia from clinical data and molecular mechanism. Front Oncol 2021;11:706030. 34650913 10.3389/fonc.2021.706030 PMC8505973

[18] Lamprianidou E , Kordella C, Kazachenka A, Zoulia E, Bernard E, Filia A, . Modulation of IL-6/STAT3 signaling axis in CD4^+^FOXP3− T cells represents a potential antitumor mechanism of azacitidine. Blood Adv 2021;5:129–42. 33570632 10.1182/bloodadvances.2020002351 PMC7805308

[19] Costantini B , Kordasti SY, Kulasekararaj AG, Jiang J, Seidl T, Abellan PP, . The effects of 5-azacytidine on the function and number of regulatory T cells and T-effectors in myelodysplastic syndrome. Haematologica 2013;98:1196–205. 23242597 10.3324/haematol.2012.074823 PMC3729899

[20] Khoury JD , Solary E, Abla O, Akkari Y, Alaggio R, Apperley JF, . The 5th edition of the World Health Organization classification of haematolymphoid tumours: myeloid and histiocytic/dendritic neoplasms. Leukemia 2022;36:1703–19. 35732831 10.1038/s41375-022-01613-1 PMC9252913

[21] Greenberg PL , Tuechler H, Schanz J, Sanz G, Garcia-Manero G, Solé F, . Revised international prognostic scoring system for myelodysplastic syndromes. Blood 2012;120:2454–65. 22740453 10.1182/blood-2012-03-420489 PMC4425443

[22] Cheson BD , Greenberg PL, Bennett JM, Lowenberg B, Wijermans PW, Nimer SD, . Clinical application and proposal for modification of the International Working Group (IWG) response criteria in myelodysplasia. Blood 2006;108:419–25. 16609072 10.1182/blood-2005-10-4149

[23] Döhner H , Wei AH, Appelbaum FR, Craddock C, DiNardo CD, Dombret H, . Diagnosis and management of AML in adults: 2022 recommendations from an international expert panel on behalf of the ELN. Blood 2022;140:1345–77. 35797463 10.1182/blood.2022016867

[24] Vakrakou AG , Paschalidis N, Pavlos E, Giannouli C, Karathanasis D, Tsipota X, . Specific myeloid signatures in peripheral blood differentiate active and rare clinical phenotypes of multiple sclerosis. Front Immunol 2023;14:1071623. 36761741 10.3389/fimmu.2023.1071623 PMC9905713

[25] Nowicka M , Krieg C, Crowell HL, Weber LM, Hartmann FJ, Guglietta S, . CyTOF workflow: differential discovery in high-throughput high-dimensional cytometry datasets. F1000Res 2017;6:748. 28663787 10.12688/f1000research.11622.1 PMC5473464

[26] Bagwell CB , Hunsberger B, Hill B, Herbert D, Bray C, Selvanantham T, . Multi-site reproducibility of a human immunophenotyping assay in whole blood and peripheral blood mononuclear cells preparations using CyTOF technology coupled with Maxpar Pathsetter, an automated data analysis system. Cytometry B Clin Cytom 2020;98:146–60. 31758746 10.1002/cyto.b.21858 PMC7543682

[27] Hao Y , Hao S, Andersen-Nissen E, Mauck WM III, Zheng S, Butler A, . Integrated analysis of multimodal single-cell data. Cell 2021;184:3573–87.e29. 34062119 10.1016/j.cell.2021.04.048 PMC8238499

[28] Korsunsky I , Millard N, Fan J, Slowikowski K, Zhang F, Wei K, . Fast, sensitive and accurate integration of single-cell data with Harmony. Nat Methods 2019;16:1289–96. 31740819 10.1038/s41592-019-0619-0 PMC6884693

[29] Aibar S , González-Blas CB, Moerman T, Huynh-Thu VA, Imrichova H, Hulselmans G, . SCENIC: single-cell regulatory network inference and clustering. Nat Methods 2017;14:1083–6. 28991892 10.1038/nmeth.4463 PMC5937676

[30] Kuleshov MV , Jones MR, Rouillard AD, Fernandez NF, Duan Q, Wang Z, . Enrichr: a comprehensive gene set enrichment analysis web server 2016 update. Nucleic Acids Res 2016;44:W90–97. 27141961 10.1093/nar/gkw377 PMC4987924

[31] Xie Z , Bailey A, Kuleshov MV, Clarke DJB, Evangelista JE, Jenkins SL, . Gene set knowledge discovery with Enrichr. Curr Protoc 2021;1:e90. 33780170 10.1002/cpz1.90 PMC8152575

[32] Huynh-Thu VA , Irrthum A, Wehenkel L, Geurts P. Inferring regulatory networks from expression data using tree-based methods. PLoS One 2010;5:e12776. 20927193 10.1371/journal.pone.0012776 PMC2946910

[33] Zhu C , Lian Y, Wang C, Wu P, Li X, Gao Y, . Single-cell transcriptomics dissects hematopoietic cell destruction and T-cell engagement in aplastic anemia. Blood 2021;138:23–33. 33763704 10.1182/blood.2020008966 PMC8349468

[34] Kornblau SM , McCue D, Singh N, Chen W, Estrov Z, Coombes KR. Recurrent expression signatures of cytokines and chemokines are present and are independently prognostic in acute myelogenous leukemia and myelodysplasia. Blood 2010;116:4251–61. 20679526 10.1182/blood-2010-01-262071 PMC4081283

[35] Sand KE , Rye KP, Mannsåker B, Bruserud O, Kittang AO. Expression patterns of chemokine receptors on circulating T cells from myelodysplastic syndrome patients. Oncoimmunology 2013;2:e23138. 23525654 10.4161/onci.23138 PMC3601181

[36] van der Leun AM , Thommen DS, Schumacher TN. CD8^+^ T cell states in human cancer: insights from single-cell analysis. Nat Rev Cancer 2020;20:218–32. 32024970 10.1038/s41568-019-0235-4 PMC7115982

[37] Zhang L , Yu X, Zheng L, Zhang Y, Li Y, Fang Q, . Lineage tracking reveals dynamic relationships of T cells in colorectal cancer. Nature 2018;564:268–72. 30479382 10.1038/s41586-018-0694-x

[38] Guo X , Zhang Y, Zheng L, Zheng C, Song J, Zhang Q, . Global characterization of T cells in non-small-cell lung cancer by single-cell sequencing. Nat Med 2018;24:978–85. 29942094 10.1038/s41591-018-0045-3

[39] Clarke J , Panwar B, Madrigal A, Singh D, Gujar R, Wood O, . Single-cell transcriptomic analysis of tissue-resident memory T cells in human lung cancer. J Exp Med 2019;216:2128–49. 31227543 10.1084/jem.20190249 PMC6719422

[40] Szabo PA , Miron M, Farber DL. Location, location, location: tissue resident memory T cells in mice and humans. Sci Immunol 2019;4:eaas9673. 30952804 10.1126/sciimmunol.aas9673 PMC6778482

[41] Kok L , Masopust D, Schumacher TN. The precursors of CD8^+^ tissue resident memory T cells: from lymphoid organs to infected tissues. Nat Rev Immunol 2022;22:283–93. 34480118 10.1038/s41577-021-00590-3 PMC8415193

[42] Li H , van der Leun AM, Yofe I, Lubling Y, Gelbard-Solodkin D, van Akkooi ACJ, . Dysfunctional CD8 T cells form a proliferative, dynamically regulated compartment within human melanoma. Cell 2019;176:775–89.e18. 30595452 10.1016/j.cell.2018.11.043 PMC7253294

[43] Chiche L , Jourde-Chiche N, Whalen E, Presnell S, Gersuk V, Dang K, . Modular transcriptional repertoire analyses of adults with systemic lupus erythematosus reveal distinct type I and type II interferon signatures. Arthritis Rheumatol 2014;66:1583–95. 24644022 10.1002/art.38628 PMC4157826

[44] Batlle E , Massagué J. Transforming growth factor-β signaling in immunity and cancer. Immunity 2019;50:924–40. 30995507 10.1016/j.immuni.2019.03.024 PMC7507121

[45] Kaech SM , Cui W. Transcriptional control of effector and memory CD8^+^ T cell differentiation. Nat Rev Immunol 2012;12:749–61. 23080391 10.1038/nri3307 PMC4137483

[46] Lee JB , Khan DH, Hurren R, Xu M, Na Y, Kang H, . Venetoclax enhances T cell-mediated antileukemic activity by increasing ROS production. Blood 2021;138:234–45. 34292323 10.1182/blood.2020009081 PMC8310428

[47] Thorsson V , Gibbs DL, Brown SD, Wolf D, Bortone DS, Ou Yang T-H, . The immune landscape of cancer. Immunity 2018;48:812–30.e14. 29628290 10.1016/j.immuni.2018.03.023 PMC5982584

[48] Winter S , Shoaie S, Kordasti S, Platzbecker U. Integrating the “immunome” in the stratification of myelodysplastic syndromes and future clinical trial design. J Clin Oncol 2020;38:1723–35. 32058844 10.1200/JCO.19.01823

[49] Yu HT , Youn J-C, Lee J, Park S, Chi H-S, Lee J, . Characterization of CD8^+^CD57^+^ T cells in patients with acute myocardial infarction. Cell Mol Immunol 2015;12:466–73. 25152079 10.1038/cmi.2014.74 PMC4496543

[50] Brenchley JM , Karandikar NJ, Betts MR, Ambrozak DR, Hill BJ, Crotty LE, . Expression of CD57 defines replicative senescence and antigen-induced apoptotic death of CD8^+^ T cells. Blood 2003;101:2711–20. 12433688 10.1182/blood-2002-07-2103

[51] Epling-Burnette PK , Painter JS, Rollison DE, Ku E, Vendron D, Widen R, . Prevalence and clinical association of clonal T-cell expansions in myelodysplastic syndrome. Leukemia 2007;21:659–67. 17301813 10.1038/sj.leu.2404590

[52] Kuo PT , Zeng Z, Salim N, Mattarollo S, Wells JW, Leggatt GR. The role of CXCR3 and its chemokine ligands in skin disease and cancer. Front Med (Lausanne) 2018;5:271. 30320116 10.3389/fmed.2018.00271 PMC6167486

[53] Maurice NJ , McElrath MJ, Andersen-Nissen E, Frahm N, Prlic M. CXCR3 enables recruitment and site-specific bystander activation of memory CD8^+^ T cells. Nat Commun 2019;10:4987. 31676770 10.1038/s41467-019-12980-2 PMC6825240

[54] Kurachi M , Kurachi J, Suenaga F, Tsukui T, Abe J, Ueha S, . Chemokine receptor CXCR3 facilitates CD8^+^ T cell differentiation into short-lived effector cells leading to memory degeneration. J Exp Med 2011;208:1605–20. 21788406 10.1084/jem.20102101 PMC3149224

[55] Rutella S , Vadakekolathu J, Mazziotta F, Reeder S, Yau T-O, Mukhopadhyay R, . Immune dysfunction signatures predict outcomes and define checkpoint blockade-unresponsive microenvironments in acute myeloid leukemia. J Clin Invest 2022;132:e159579. 36099049 10.1172/JCI159579 PMC9621145

[56] Wenk C , Garz A-K, Grath S, Huberle C, Witham D, Weickert M, . Direct modulation of the bone marrow mesenchymal stromal cell compartment by azacitidine enhances healthy hematopoiesis. Blood Adv 2018;2:3447–61. 30518537 10.1182/bloodadvances.2018022053 PMC6290099

[57] Platzbecker U , Porta MGD, Santini V, Zeidan AM, Komrokji RS, Shortt J, . Efficacy and safety of luspatercept versus epoetin alfa in erythropoiesis-stimulating agent-naive, transfusion-dependent, lower-risk myelodysplastic syndromes (COMMANDS): interim analysis of a phase 3, open-label, randomised controlled trial. Lancet 2023;402:373–85. 37311468 10.1016/S0140-6736(23)00874-7

[58] Overacre-Delgoffe AE , Chikina M, Dadey RE, Yano H, Brunazzi EA, Shayan G, . Interferon-γ drives T_reg_ fragility to promote anti-tumor immunity. Cell 2017;169:1130–41.e11. 28552348 10.1016/j.cell.2017.05.005 PMC5509332

[59] Boukhaled GM , Harding S, Brooks DG. Opposing roles of type I interferons in cancer immunity. Annu Rev Pathol 2021;16:167–98. 33264572 10.1146/annurev-pathol-031920-093932 PMC8063563

[60] Chiappinelli KB , Strissel PL, Desrichard A, Li H, Henke C, Akman B, . Inhibiting DNA methylation causes an interferon response in cancer via dsRNA including endogenous retroviruses. Cell 2015;162:974–86. 26317466 10.1016/j.cell.2015.07.011 PMC4556003

[61] Roulois D , Yau HL, Singhania R, Wang Y, Danesh A, Shen SY, . DNA-demethylating agents target colorectal cancer cells by inducing viral mimicry by endogenous transcripts. Cell 2015;162:961–73. 26317465 10.1016/j.cell.2015.07.056 PMC4843502

[62] Kazachenka A , Young GR, Attig J, Kordella C, Lamprianidou E, Zoulia E, . Epigenetic therapy of myelodysplastic syndromes connects to cellular differentiation independently of endogenous retroelement derepression. Genome Med 2019;11:86. 31870430 10.1186/s13073-019-0707-x PMC6929315

[63] Ohtani H , Ørskov AD, Helbo AS, Gillberg L, Liu M, Zhou W, . Activation of a subset of evolutionarily young transposable elements and innate immunity are linked to clinical responses to 5-azacytidine. Cancer Res 2020;80:2441–50. 32245794 10.1158/0008-5472.CAN-19-1696 PMC7507765

[64] Heinhuis KM , Ros W, Kok M, Steeghs N, Beijnen JH, Schellens JHM. Enhancing antitumor response by combining immune checkpoint inhibitors with chemotherapy in solid tumors. Ann Oncol 2019;30:219–35. 30608567 10.1093/annonc/mdy551

[65] Miller BC , Sen DR, Al Abosy R, Bi K, Virkud YV, LaFleur MW, . Subsets of exhausted CD8^+^ T cells differentially mediate tumor control and respond to checkpoint blockade. Nat Immunol 2019;20:326–36. 30778252 10.1038/s41590-019-0312-6 PMC6673650

[66] Shallis RM , Bewersdorf JP, Gowda L, Podoltsev NA, Prebet T, Gore SD, . Immune checkpoint inhibitor therapy for acute myeloid leukemia and higher-risk myelodysplastic syndromes: a single-center experience. Blood 2019;134(Suppl 1):1330.

